# A User-Friendly Model for Spray Drying to Aid Pharmaceutical Product Development

**DOI:** 10.1371/journal.pone.0074403

**Published:** 2013-09-09

**Authors:** Niels Grasmeijer, Hans de Waard, Wouter L. J. Hinrichs, Henderik W. Frijlink

**Affiliations:** Department of Pharmaceutical Technology and Biopharmacy, University of Groningen, Groningen, The Netherlands; University of Illinois at Chicago, United States of America

## Abstract

The aim of this study was to develop a user-friendly model for spray drying that can aid in the development of a pharmaceutical product, by shifting from a trial-and-error towards a quality-by-design approach. To achieve this, a spray dryer model was developed in commercial and open source spreadsheet software. The output of the model was first fitted to the experimental output of a Büchi B-290 spray dryer and subsequently validated. The predicted outlet temperatures of the spray dryer model matched the experimental values very well over the entire range of spray dryer settings that were tested. Finally, the model was applied to produce glassy sugars by spray drying, an often used excipient in formulations of biopharmaceuticals. For the production of glassy sugars, the model was extended to predict the relative humidity at the outlet, which is not measured in the spray dryer by default. This extended model was then successfully used to predict whether specific settings were suitable for producing glassy trehalose and inulin by spray drying. In conclusion, a spray dryer model was developed that is able to predict the output parameters of the spray drying process. The model can aid the development of spray dried pharmaceutical products by shifting from a trial-and-error towards a quality-by-design approach.

## Introduction

Pharmaceutical product development can be a costly and time consuming process. Although fairly simple processes allow researchers to base their development on a trial-and-error approach, more complex processes will quickly increase the required number of experiments to unfeasible heights. To allow products to be developed with reasonable resources on more complex processes, a shift towards a quality-by-design approach is desired. Even more so, a quality-by-design approach can not only improve the development stage, but will also tremendously aid in the quality control of the end product since it forces researchers to acquire a more detailed and fundamental understanding of the processes used for production. This is also the main reason why the FDA and EMA advocate the use of quality-by-design in drug development, and a framework for this approach has been developed in the ICH guidelines Q8, Q9, and Q10 [1], [2]. Moving from a trial-and-error approach to a quality by design approach, requires the development of a model of the production process linking variables to critical quality attributes of the final product. The use of such a process model can aid pharmaceutical product development in several ways.

First, determining the design space can be considerably more efficient, since a model allows output parameters to be calculated without performing a considerable amount of experimental work. One might think that the time required to develop such a model will hardly ever compensate the time gained during product development by shifting from a trial-and-error to a quality-by-design approach. Indeed, when a model would be specifically designed for one product, the time required to develop the model could easily be longer than the time gained. On the other hand, the model could still improve the quality of the product and could therefore be advantageous. However, a model that is more generally applicable to the process or even multiple processes would be advantageous, since such a model would only have to be developed once and can be used for other products and future research as well.

Secondly, the use of even a basic model can greatly increase the understanding of a process. Although the user may not have taken part in the development of the model, the use of the model does allow one to quickly see effects of changes in various parameters on the output of the process. However, the detail and number of affected parameters does depend on the complexity of the model.

Finally, a good model can also give additional relevant process information that is not provided inline during processing. There can be many parameters that are not measured inline, but they can be very useful for the researcher developing a process. Although most of these parameters can be determined offline, this would require additional experiments. Therefore, although a basic model can be developed to provide the user with just one critical output parameter of a process, other parameters that are usually not measured inline can be calculated and added to the result, thereby expanding the usefulness of the model. The addition of otherwise unknown output parameters can be invaluable in the development stage of a pharmaceutical product.

A process that can significantly benefit from being modeled is spray drying. The many input and output parameters make it a complex process to optimize by trial-and-error during product development. Furthermore, several process parameters and product properties can be very difficult to measure inline. Several papers have been published on the development of a spray dryer model to facilitate a shift from trial-and-error to quality-by-design [3]–[6]. Unfortunately, the models that are presented in these studies are often either based on very complex computational fluid dynamics (CFD) [7] or they are developed in expensive and specialized software [3]. Although these models are useful for many applications, the details of the models and the complexity of the software far exceeds that required for standard pharmaceutical product development. Therefore, a pharmaceutical scientist cannot easily use or even understand the models that were studied, which does not add to a fundamental understanding of the modeled process. In that respect, a model that is developed in software that is common amongst pharmaceutical scientists would be preferred. In addition, it would be even more useful if said model was easily adaptable for different spray dryers and individual user needs.

Therefore, in this study, common mass and energy balances were used as a basis to develop a user-friendly spray dryer model that can aid in the development of spray dried products. The model will be presented in such a way, that it can be used by those less familiar with spray drying and specialized software, and that it can be adapted for different spray dryers. Furthermore, public access to the model is ensured by developing the model in an open source software package and making it available through an open access journal (File S1–S3).

As an example we will use spray drying of an aqueous trehalose and aqueous inulin solution. Spray drying of these disaccharides is of interest in modern pharmaceutics, since both have been shown to be good stabilizers for biopharmaceuticals such as therapeutic peptides, proteins, or vaccines [8]–[14]. Using the adequate drying process is of paramount importance here since stabilization of the incorporated biopharmaceutical will in general only be obtained when the sugar is in the glassy state. To obtain glassy material from the drying process requires specific and well controlled process conditions and adequate process understanding. This makes the example of trehalose and inulin interesting for many development scientists. However, the aim of the model is not to determine the influence of excipients on the outcome of the spray drying process, but rather the influence of the process conditions on the final product. Therefore, the example of glassy sugar production, which can be applied to protein stabilization, is merely used to show the application of the model in the specific field of protein stabilization, where glassy sugars are desired. In fact, the spray dryer model can be applied to numerous spray drying applications due to the general setup of the model. It will, however, not predict whether a protein will be stabilized, as it will also depend on the type of sugar used, but rather the optimal spray drying conditions to stabilize a protein.

The model development will be divided into three separate stages. First, a basic model will be developed that will enable us to calculate the spray dryer outlet temperature. Then, for the purpose of obtaining glassy sugars by spray drying, the model will be extended to include a relative humidity calculation, which is an essential parameter. Finally, this extended model will be used to predict whether glassy trehalose and inulin can be obtained successfully by spray drying at specific inlet conditions.

## Materials and Methods

### Materials

Trehalose was obtained from Cargill B.V. (Amsterdam, The Netherlands). Inulin was kindly provided by Sensus (Roosendaal, The Netherlands) and had a degree of polymerization of 11. All experiments were performed with millipore water, type 1.

### Spray Drying Process

Model validation and sample preparation were done by performing several spray drying experiments with a B-290 spray dryer in conjunction with a high performance cyclone and a B-295 dehumidifier (Büchi Labortechnik AG, Flawil, Switzerland). All results were obtained with the spray dryer in closed loop configuration. Furthermore, spray drying experiments that included a liquid feed flow were performed using water only, except for the experiments with trehalose or inulin, which were performed using an aqueous solution containing 2.5% w/v trehalose or inulin. For model fitting, validation, and relative humidity measurements, the inlet temperature was varied between 50°C and 200°C, the liquid feed flow between 0 mL/min and 4.9 mL/min, and the aspirator flow between 50% and 100%. Trehalose and inulin were spray dried at a constant inlet temperature of 70°C, while the aspirator flow was set at 100%. The liquid feed flow for the aqueous trehalose solution was set at 4.1 and 5.1 mL/min, and for the aqueous inulin solution at 4.5 and 5.7 mL/min, using a syringe pump. The atomizing airflow was kept constant for all experiments at 600 L_n_/hr, which corresponds to a setting of 50 mm (normal liter (L_n_) is the volume at 0°C and 1 atm). Specific spray dryer settings used for fitting, validation, and measuring the relative humidity at the outlet are shown in Table 1 and 2. During the spray drying experiments, equilibrium of outlet conditions was assumed to exist when the temperature did not change more than 0.5°C during 5 minutes.

**Table 1:** Spray dryer settings used for model fitting and validation.

Inlet temperature (°C)	Aspirator flow^a^ (m^3^_n_/hr)	Liquid feed flow (mL/min)
50	12	0
50	22	0
100	12	0
100	22	0
100	22	1.3
100	22	2.7
100	22	3.6
100	22	4.9
150	12	0
150	22	0
150	22	1.3
150	22	2.7
150	22	3.6
150	22	4.9
200	12	0
200	22	0
200	22	1.3
200	22	2.7
200	22	3.6
200	22	4.9

a Aspirator flow of 12 and 22 m^3^_n_/hr corresponded to a setting of 50% and 100%, respectively (determined with the flow rate - pressure drop relationship over the cyclone and filter as described in the flow rate – pressure drop relationship section in materials and methods).

**Table 2:** Spray dryer settings used for relative humidity measurements.

Inlet temperature (°C)	Aspirator flow^a^ (m^3^_n_/hr)	Liquid feed flow (mL/min)
50	22	0
50	22	1.3
50	22	2.7
50	22	3.6
70	12	0
70	12	1.3
70	12	2.7
70	22	0
70	22	1.3
70	22	2.7
70	22	3.6
70	22	4.9
90	12	0
90	12	1.3
90	12	2.7
90	12	3.6
90	22	0
90	22	1.3
90	22	2.7
90	22	3.6
90	22	4.9

a Aspirator flow of 12 and 22 m^3^_n_/hr corresponded to a setting of 50% and 100%, respectively (determined with the flow rate - pressure drop relationship over the cyclone and filter as described in section 2.3).

### Flow Rate – pressure Drop Relationship

Since the aspirator flow of the B-290 spray dryer is expressed in percentage, the mass flow of the system had to be determined with respect to the given percentage in order to use the aspirator flow in the model. To minimize the influence on the spray dryer process, the aspirator flow was determined using the flow rate - pressure drop relationship, where the flow rate through a system is related to the square root of the pressure drop over the same system. The pressure drop over the cyclone and the filter was measured with a HBM PD1 differential pressure transducer in conjunction with a HBM MC2A measuring converter (Hottinger Baldwin Messtechnik, Darmstadt, Germany) at flow rates between 0 and 150 L_n_/min, after which the slope of the relation between the flow rate and the square root of the pressure drop could be determined (*R*). The flow rate through the cyclone and the filter was determined using a Brooks 5863S mass flow meter (Brooks Instruments B.V., Ede, The Netherlands). Subsequently, the pressure drop (*Δp*) across the cyclone and the filter was measured inside the spray dryer at aspirator settings between 50 and 100%. The aspirator flow inside the spray dryer (*Q_V.g_*) with respect to the spray dryer setting was then calculated (Eq. 1). The slope and intercept of the linear relationship between the aspirator setting in percentage and actual flow rate were used in the model. Because the pressure drop of the high performance cyclone may differ between copies, the flow rate – pressure drop relationship will have to be determined separately for every cyclone used.
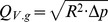
(1)


### Relative Humidity

Relative humidity measurements were performed using a Testo 650 handheld device with a standard climate sensor (Testo B.V., The Hague, The Netherlands). The sensor had a relative humidity range of 0% to 100% (±2%) and a temperature range of −20°C to +70°C (±0.5°C), limiting the inlet temperature to a maximum of 90°C with the chosen liquid feed flow and aspirator flow (Table 2). Measurements were done directly behind the outlet temperature sensor of the B-290 spray dryer.

### Differential Scanning Calorimetry (DSC)

Modulated DSC measurements were done with a DSC 2920 differential scanning calorimeter (TA Instruments, New Castle, United States). Humidified spray dried trehalose and inulin samples were prepared by storing the samples at a relative humidity of 22%, 33%, and 52% in a desiccator over a saturated aqueous solution of CH_3_COOK, MgCl_2_.6H_2_O, and Na_2_Cr_2_O_7._2H_2_O, respectively, or at 45%, and 60% in a climate chamber for 1–3 weeks. Humidified samples were weighed in closed aluminum pans, then cooled to −20°C, and finally heated at a rate of 2°C/min with a modulation period of 60 seconds and amplitude of 0.316°C. The glass transition temperature was taken as the inflection point of the transition in the reversing heat flow versus temperature curve.

### Dynamic Vapor Sorption (DVS) analysis

The water sorption isotherms of spray dried trehalose and inulin were measured at ambient pressure and 25°C using a DVS-1000 water sorption instrument (Surface Measurement Systems Limited, London, UK). The moisture content was determined at relative humidity’s ranging from 0–90% in 10% increments, for a sample with an initial mass of approximately 10 mg. After subjecting the samples to the specified humidity, equilibrium was assumed when the change in mass was less than 0.9 µg during 10 minutes.

### Model Development

**Figure 1:**
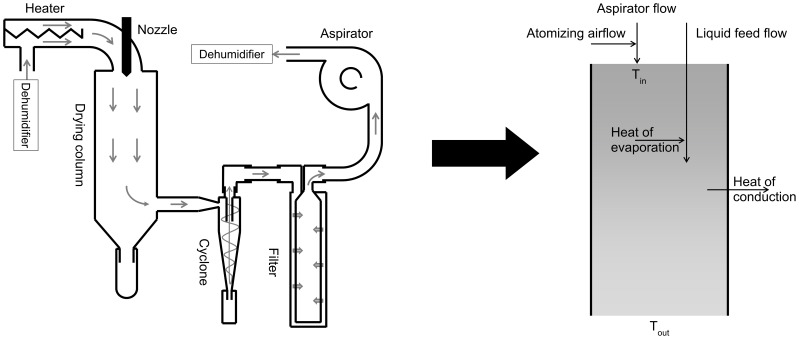
Schematic representation of a spray dryer (left) and the simplified spray dryer model (right). Output variables include but are not restricted to the outlet temperature (*T_out_*).

The spray dryer model was developed and tested using both commercial and open source spreadsheet software packages, namely: Office 2003 and 2010 (Microsoft), Libreoffice 3.4 (The Document Foundation), and OpenOffice.org 3.3 (The Apache Software Foundation). The model was based on the B-290 spray dryer, which was simplified for the development of the model, as shown schematically in Figure 1.

The entire spray dryer was considered to be a cylinder (right of Figure 1) with a diameter and wall thickness that could be measured directly from the device used by the researcher. Although the actual spray dryer is more complex than a simple cylinder, inner flow characteristics are not considered and the drying gas is considered to be continuously and ideally mixed. Due to these assumptions, the main parameters that determine the outlet temperature are mainly limited to the surface area and properties of the wall and surrounding medium. Therefore, the complex shaped spray dryer can be modeled as a straight cylinder.

Whereas the inlet of the spray dryer consists of three separate streams: the atomizing airflow, aspirator airflow, and liquid feed flow, the outlet consists of one single gas stream. Using several input parameters, the outlet temperature can be calculated using basic thermodynamic equations. As shown in Figure 1, the outlet temperature is determined by the heat loss through both conduction and evaporation. The heat loss through conduction (*Q_h.con_*) can be calculated using a basic heat transfer equation for flat surfaces, whereas the heat loss through evaporation (*Q_h.evap_*) is straightforward, as shown in Eq. (2) and Eq. (3) respectively.
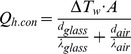
(2)

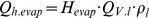
(3)


Where *ΔT_w_* is the log mean temperature difference across the wall over the entire length of the spray dryer, *A* is the surface area of the wall, *d_glass_* is the thickness of the wall, *d_air_* is the boundary layer of air on the outside, *λ_glass_* and *λ_air_* are the heat conductivity of the glass and air respectively, *H_evap_* is the heat of evaporation of the liquid, *Q_V.l_* is the liquid feed flow, and *ρ_l_* is the density of the liquid.

Except for *ΔT_w_* and *d_air_,* all parameters are known. The unknown parameter, *ΔT_w_*, can be calculated using Eq. (4).
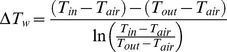
(4)


Where *T_in_* and *T_out_* are the temperature of the heated drying air at the spray dryer inlet and outlet respectively, and *T_air_* is the temperature of the ambient air, which is assumed to be constant. The unknown input parameter, *d_air_*, was used as a fitting parameter, to match the output values of the model to experimentally determined values that were obtained by running the spray dryer under various conditions.

Finally, the outlet temperature can be calculated by subtracting the heat flow due to evaporation and conduction from the heat capacity of the aspirator flow, as shown in Eq. (5).
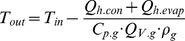
(5)


**Figure 2:**
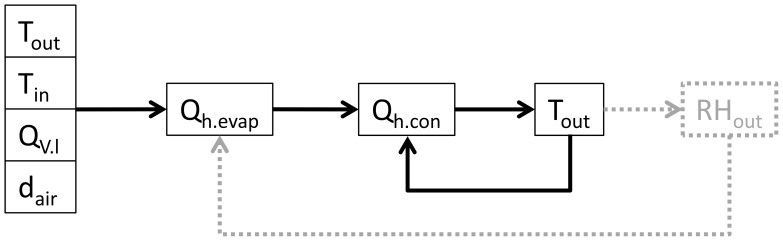
Overview of iteration steps in our spray dryer model. Details are left out for clarity. Model expansion for relative humidity at the spray dryer outlet (*RH_out_*) will be discussed in the results.

Where *C_p.g_* is the heat capacity if the drying gas under constant pressure, *Q_V.g_* is the drying gas flow rate, and *ρ_g_* is the density of the gas. However, because *ΔT_w_* (and thus heat loss due to conduction, Q_h.con_) is dependent on the outlet temperature of the spray dryer, the calculation was repeated, or iterated, until the output was constant (Figure 2).

As shown in Figure 2, the relative humidity at the outlet (*RH_out_)* can also be calculated when the outlet temperature is known. The relative humidity can be calculated with the Antoine equation and the ideal gas law (Eq. (6) and Eq. (7) respectively).
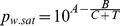
(6)

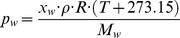
(7)


Where *p_w.sat_* is the saturated water vapor pressure, *A*, *B,* and *C* are the Antoine constants of water (10.20, 1730.63, and 233.43, respectively [15]), *T* is the temperature (°C), *p_w_* is the partial water vapor pressure (Pa), *x_w_* is the specific humidity, *ρ* is the density of air, *R* is the gas constant, and *M_w_* is the molecular mass of water. Since all the parameters are known during the iterated calculation in the spreadsheet software, the relative humidity, which is defined as *p_w_*/*p_w.sat_*·100, can be calculated.

## Results

### Model Basis

**Figure 3:**
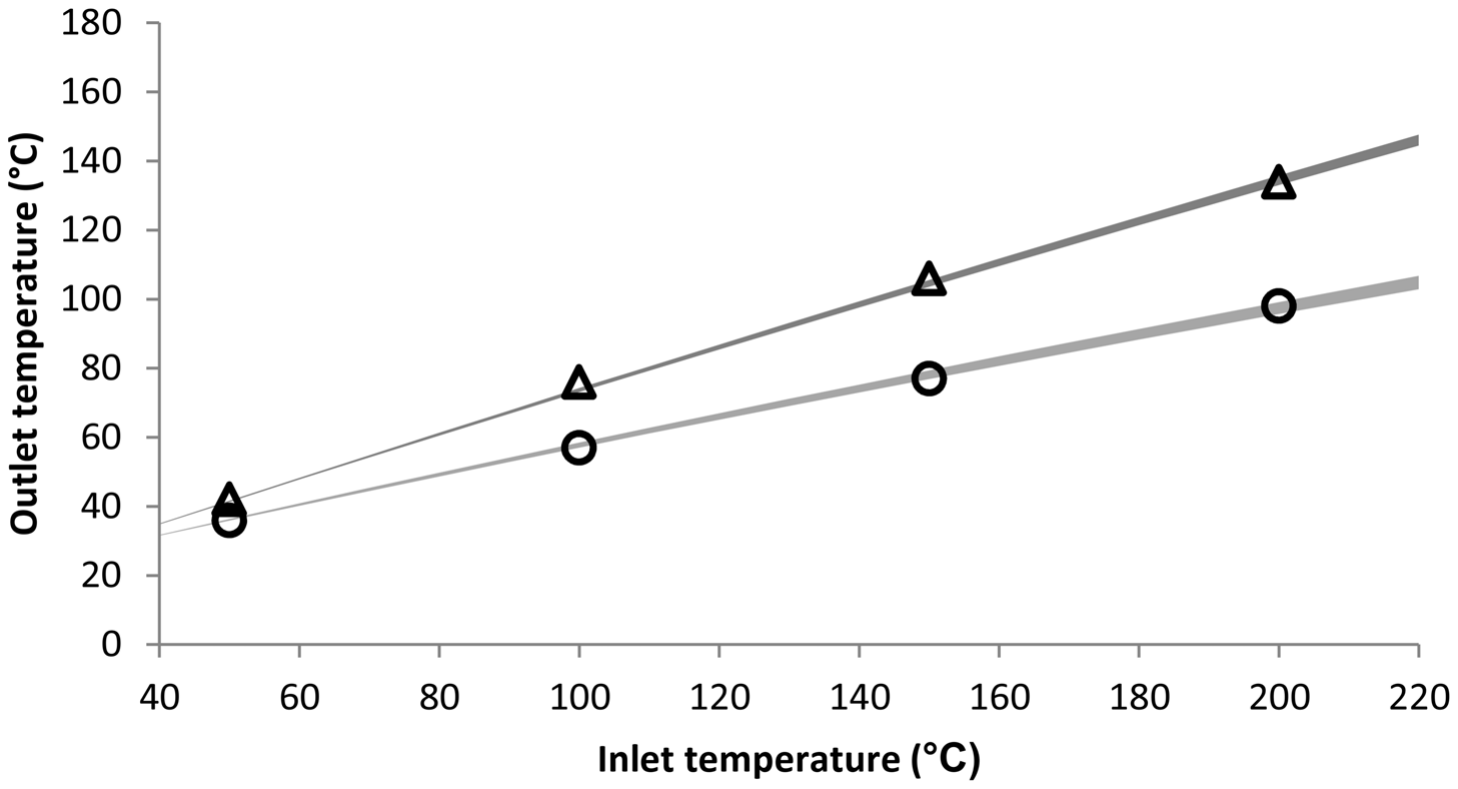
Modeled (grey) and experimental (black) outlet temperature used to determine *d_air_*. Aspirator flow was set at either 12 m^3^_n_/hr (circle) or 22 m^3^_n_/hr (triangle), while the liquid feed flow was kept constant at 0 mL/min. Thickness of the lines indicate the 95% confidence interval of the modeled outlet temperature.

A basic model was developed, as described in the model development section in materials and methods, using only freely available software. First the model was fitted to experimental values by running the spray dryer under various conditions to determine the value of the fitting parameter, *d_air_*. Since the fitting parameter, *d_air_*, solely determines the heat loss due to conduction and not due to evaporation, the experiments were conducted without a liquid feed. In other words, only a heated gas flow through the spray dryer was considered. By using the least squares method on the modeled outlet temperature and experimental outlet temperature, the optimum value for *d_air_* was found to be 1.97 mm. With this value, the modeled outlet temperature matched the experimental outlet temperature well for all settings, with a difference ranging between −2.5 and +1.5°C (Figure 3).

For the confidence assessment of the fitting parameter, *d_air_*, a protocol described by Kemmer et al was used [16]. Based on this protocol, the 95% confidence interval of *d_air_* was calculated to be between 1.91 and 2.03 mm. The thickness of the line in Figure 3 indicates the range of modeled outlet temperatures corresponding to this 95% confidence interval. Furthermore, the mean difference of the modeled and experimental values (modeled values minus experimental values) was −0.3°C, indicating a slight bias of the model towards a lower outlet temperature. Finally, the mean absolute difference (the mean of the absolute difference between modeled and experimental values) was found to be 0.9°C, which indicates a good precision.

**Figure 4:**
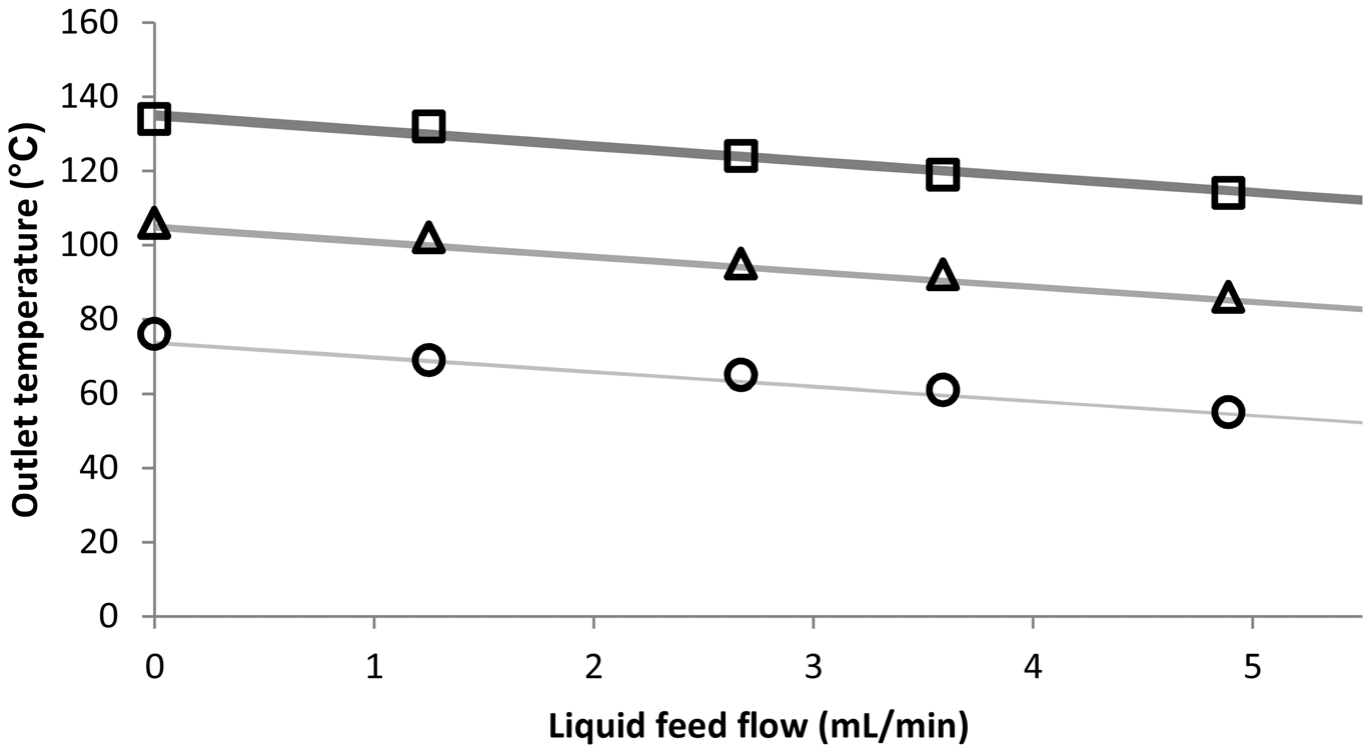
Modeled (grey) and experimental (black) outlet temperature. **Inlet temperature was set at 100°C (circle), 150°C (triangle), or 200°C (square).** Aspirator flow was kept constant at 22 m^3^_n_/hr. Thickness of the lines indicate the 95% confidence interval of the modeled outlet temperature.

Subsequently, the fitted model was validated. This was done by comparing the model output to several spray drying measurements that included a liquid feed flow (Figure 4). The modeled outlet temperature was found to match the experimentally determined outlet temperature very well. Both at a high and low inlet temperature of 200°C and 100°C, respectively, the outlet temperature matched the experimentally determined outlet temperature even at the highest liquid feed flow of 5 mL/min, with a difference ranging between −2.5 and +1°C. It should be noted, however, that some of the experimentally determined values lie outside the 95% confidence interval, which is most likely due to the low number of data points used for fitting the model. Despite this deviation, the mean difference was found to be −0.8°C, indicating a slight underestimation of the modeled outlet temperature. In addition, the mean absolute difference was 1.1°C, which shows that the precision of the model is high.

Although the model was able to predict the outlet temperature of the B-290 spray dryer very well, it would be even more useful if the model could also be used for other spray dryers. Therefore, an attempt was made to adapt the model for another type of spray dryer. Although the B-290 spray dryer complicated the development, due to the necessary conversion of aspirator setting in percentage to actual volume and mass flow rate, it was possible to adapt the model for a B-90 spray dryer, which reports the aspirator flow in L/min. Although not implied by the name, the B-90 spray dryer is very different from the B-290 spray dryer. Not only does the nozzle consist of an ultrasonic sprayhead, without the atomizing airflow, but the B-90 spray dryer also uses an electrostatic collector instead of a cyclone. In addition, the spray dryer is shaped like a cylinder instead of the more complex system of components that composes the B-290 spray dryer.

The model was fitted to the spray dryer by simply measuring the dimensions of the drying column (length, diameter, glass thickness) and performing 6 spray drying experiments without a liquid feed flow. Spray drying was performed at an inlet temperature of either 50, 90, or 120°C and an aspirator flow of either 85 or 165 L/min. It was found that the modeled outlet temperature again matched the experimentally determined outlet temperature very well for all settings, with a difference between ±2°C (data not shown). The mean difference was 0°C, indicating that there is no bias of the model, whereas the precision of the model was similar to the fit of the B-290 spray dryer with a mean absolute difference of 1.3°C.

### Model Extension

An interesting application of spray drying is the stabilization of biopharmaceuticals with sugar glasses. When a biopharmaceutical is incorporated in a matrix of a glassy sugar, it can retain its conformation for prolonged periods in the dry state. The conformation of the biopharmaceutical can be retained due to several mechanisms. Although various mechanisms are proposed to play a role, one of the most often considered hypothesis is the vitrification theory [13], [17]–[19]. The vitrification theory states that the biopharmaceutical is immobilized when it is incorporated in a sugar. Since most degradation pathways require molecular mobility, the degradation rate is strongly reduced. To immobilize the biopharmaceutical, it is important that the sugar can accommodate the irregular surface of the biopharmaceutical. Therefore, the sugar should be in the amorphous state and not in the crystalline state [20]. More specifically, the amorphous sugar should be in the glassy state and not in the rubbery state for three important reasons. Firstly, in the glassy state the translational molecular mobility is low, which is required for vitrification, whereas in the rubbery state the translational molecular mobility is relatively high, which facilitates degradation of the biopharmaceutical [21]. Secondly, in the rubbery state the sugar can easily crystallize. Thirdly, what is highly relevant for the spray drying process is that in the rubbery state the sugar also tends to be sticky. As a consequence, the rubbery sugar is more likely to stick to the cyclone wall, reducing the yield of the product [22]. Therefore, it is important that the glass-rubber transition temperature of the product is higher than the surrounding temperature.

The relative humidity of the drying air is an important parameter during the production of amorphous sugars by spray drying. Not only does humid air cause the water droplets to evaporate slower, it also lowers the glass transition temperature of amorphous sugars as adsorbed moisture acts as a plasticizer. Therefore, usually, a dry product with a moisture content as low as possible is aimed for. Although the relative humidity, and thus the product moisture content, can be lowered by simply increasing the temperature, exposing the material to excessive temperatures should in general be avoided to prevent thermal degradation. To find the optimum balance between relative humidity and outlet temperature, while also maximizing the throughput, optimization is required. Therefore, any information on relative humidity prior to spray drying can be highly relevant to the development scientist. Unfortunately, in most commercially available lab-scale spray dryers a relative humidity measurement is not included and would therefore be a desirable addition to a spray dryer model. Therefore, the basic model was extended to include the relative humidity in order to increase the usefulness of the model, as described in the model development section in materials and methods.

**Figure 5:**
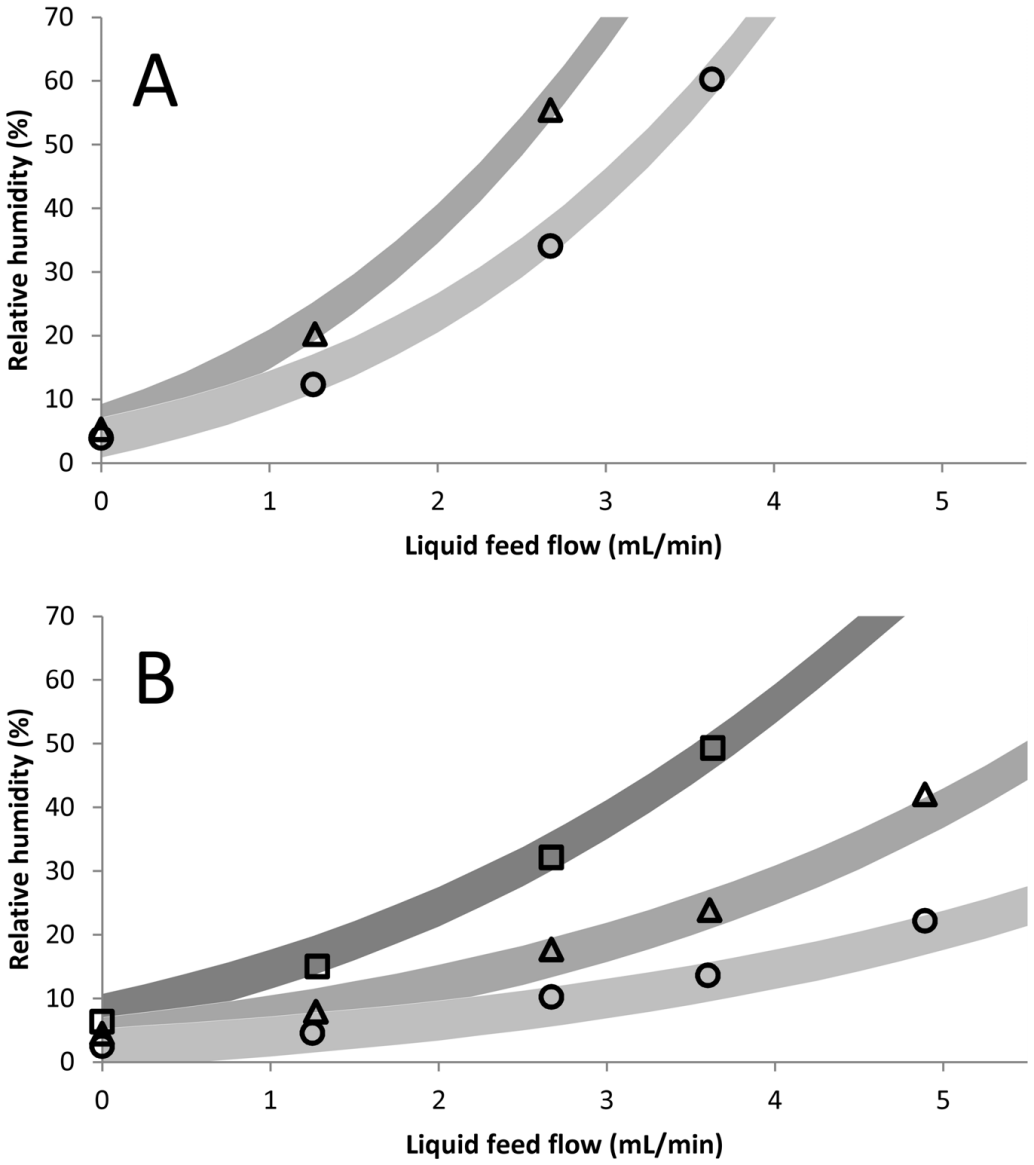
Modeled (grey) and experimental (black) relative humidity. Measurements were done at an inlet temperature of 90°C (circle), 70°C (triangle), or 50°C (square). Results shown at the top (A) were obtained with an aspirator flow of 12 m^3^_n_/hr and the results on the bottom (B) with 22 m^3^_n_/hr. Thickness of the lines indicate the 95% confidence interval of the modeled relative humidity (3.1% RH).

To validate the results of the extended model, the relative humidity at the spray dryer outlet was measured at various spray dryer settings (Table 2), and compared to the relative humidity predicted by the model (Figure 5). During the measurements, the reported temperature of the relative humidity sensor was only slightly higher than the temperature that was reported by the spray dryer itself (1–3°C). Therefore, the fitting parameter, *d_air_,* was not adjusted to fit the model to the temperature reported by the relative humidity sensor, but kept at 1.97 mm. This resulted in a slight underestimation of the modeled outlet temperature, averaging around −2°C, when compared to the outlet temperature measured with the relative humidity probe. On the other hand, the modeled outlet temperature matched the outlet temperature reported by the B-290 spray dryer very well with a mean difference of +0.2°C, and a mean absolute difference of 1.3°C.

The relative humidity was predicted well by the model. The difference between the modeled and experimentally determined relative humidity ranged between −3.5 and +2.1% RH, with the largest difference at a liquid feed flow of 4.9 mL/min. However, when calculating the modeled relative humidity according to the 95% confidence interval that was determined based on the fitting results, it was found that most of the experimental relative humidity values were outside this confidence interval (data not shown). In addition, calculating the confidence interval based on the relative humidity data did not yield a correct confidence interval either (data not shown). Therefore, a more general approach was applied as described by Brown, by which the 95% confidence interval is determined directly from the modeled and experimental value [23]. With this method a 95% confidence interval of 3.1% RH was calculated, which appears to fit all the experimental values (Figure 5). Furthermore, the mean difference between the modeled and experimental relative humidity was 0% RH, indicating no bias of the model. Finally, the mean absolute difference was 1.2% RH, which is considered precise.

### Model Application

To show the applicability of the model we took the example of trehalose and inulin, both suitable excipients for stabilization of biopharmaceuticals during spray drying. Although the glass transition temperature of trehalose and inulin are relatively high (121°C and 130°C, respectively), it can be greatly reduced by adsorbed moisture, as discussed in the model extension section in the results. Therefore, knowledge of the hygroscopicity and quantification of the reduction of the glass transition temperature due to adsorbed moisture is key to understanding the outcome of the spray drying process. Therefore, the model was used in conjunction with DVS and DSC analyses to determine the optimal settings for spray drying both a trehalose and an inulin solution.

The glass transition temperature dependence on the moisture content can be described by the Gordon-Taylor equation (Eq. 8), which describes the relation between the composition of an ideal and homogeneous mixture consisting of two components (with mass fractions *w_s_*_,_ and *w_w_*) and its glass transition temperature (*T_g_*) [24]. Besides the mass fraction of the components, the glass transition temperature of the mixture is also dependent on the glass transition temperature of the individual components (*T_g.s_*_,_ and *T_g.w_*) and a component-dependent Gordon-Taylor constant (*k_sw_*). The subscripts *s*, and *w* are used for sugar, and water respectively. To calculate the glass transition temperature of a trehalose-water and inulin-water mixture with the Gordon-Taylor equation, the glass transition temperature of trehalose and inulin, the Gordon-Taylor constants and the mass fraction of water were determined.
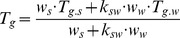
(8)


The glass transition temperature of trehalose and inulin were determined with DSC and found to be 121°C and 130°C, respectively. For water, a glass transition temperature of −109°C was used, which is the average of recently published values [25]–[27]. Although this value is substantially higher than the conventionally accepted value of −137°C [25]–[27], our calculations indicated that the choice of either of these glass transition temperatures of water did not have a large influence on the calculated glass transition temperature of the final samples.

The Gordon-Taylor constant, *k_sw_*, for a trehalose-water and inulin-water mixture was determined by fitting the Gordon-Taylor equation with glass transition temperatures of humidified sugar glasses measured with DSC. The mass fraction of water (*w_w_*) of these humidified sugar glasses was determined with DVS analysis. The Gordon-Taylor constant for the trehalose-water and inulin-water mixture were found to be 7.90 and 7.40, respectively. The Gordon-Taylor constant for a trehalose-water and inulin-water mixture was higher than values found in literature (i.e. 5.2, 6.5, and 7.3 and between 5.9 and 6.4, respectively), due to the higher glass transition temperature of water we used [8], [28]–[30].

The mass fraction of water, *w_w_*, at a spray dryer setting of choice, was determined by relating the relative humidity output of the model to DVS data of trehalose or inulin. Thereby, it is assumed that the moisture content of the spray dried sugar is in equilibrium with the outlet conditions of the spray dryer.

Depending on the requirements of the product and process, the inlet temperature, liquid feed flow and aspirator can be varied in the model to find the optimum settings, where the glass transition temperature of the sugar is higher than the outlet temperature of the spray dryer. An example is shown, where the liquid feed flow is changed slightly to determine the effect on the yield of spray dried trehalose and inulin (Table 3). Assuming that the moisture content is in equilibrium with the outlet conditions, at a liquid feed flow of 5.1 mL/min the glass transition temperature of trehalose (25°C) is expected to be below the outlet temperature (35°C). A liquid feed flow of 5.7 mL/min was used to obtain the same difference between the glass transition temperature of inulin (22°C) and the outlet temperature (32°C). Because, under these conditions, the glass transition temperature is lower than the outlet temperature, trehalose and inulin are expected to be in a rubbery, sticky, state. In contrast, at a liquid feed flow of 4.1 mL/min, a lower relative humidity is expected, resulting in a glass transition temperature of trehalose (49°C) above the outlet temperature (39°C). A liquid feed flow of 4.5 mL/min was used to obtain the same difference between the glass transition temperature of inulin (47°C) and the outlet temperature (37°C). Because, under these conditions, the glass transition temperature is higher than the outlet temperature, trehalose and inulin are expected to be in a glassy, non-sticky state.

**Table 3:** Trehalose and inulin yield depending on spray drying conditions.^a^

	Trehalose		Inulin	
Liquid feed flow (mL/min)	5.1	4.1	5.7	4.5
RH model (%)	41	29	52	34
Tout model (°C)	35	39	32	37
Estimated Tg (°C)^b^	25±5	49±7	22±5	47±4
Measured Yield (%)	4	68	8	75

a The inlet temperature was set at 70°C and the aspirator was set at 22 m^3^_n_/hr (100%).

b The margin of error for the estimated Tg is based on the 95% confidence interval of the modeled relative humidity (3.1% RH).

It was found that the experimental observations agreed with the modeled conditions. At a liquid feed flow of 5.1 and 5.7 mL/min, when the amorphous powder was expected to be in its sticky rubbery state, the yield was very low (4 and 8% of trehalose and inulin, respectively) and the cyclone wall was completely covered with powder. However, at a liquid feed flow of 4.1 and 4.5 mL/min, when the amorphous powder was expected to be in its glassy state, the yield was much higher (68 and 75% of trehalose and inulin, respectively) and hardly any powder was visible on the cyclone wall after spray drying. DSC confirmed that both trehalose and inulin were in an amorphous state.

## Discussion

A spray dryer model is presented that is developed in an open source spreadsheet program and made freely available, which enables the use of the model by virtually everyone. Additional information on the use of the model is also made available online (File S1–S3). The development of this model was explained to provide the basic knowledge required to understand the model. Furthermore, the model allows the user to make adaptations in case one’s process deviates from the spray drying process that is used to develop the model. Such a user-friendly model for spray drying can aid pharmaceutical product development by shifting from a trial-and-error to a quality-by-design approach. We confirmed this by the successful application of the model to spray drying of trehalose and inulin.

The model was developed while keeping in mind that the end user may not be familiar with mathematical software programs, energy and mass balances or even the spray drying process. Therefore, it was decided to develop the model in an open source spreadsheet program. One could argue that the use of a program such as Mathcad or an open source alternative would be more appropriate due to the visibility of the symbolic equations. Indeed, a large disadvantage of spreadsheet software is that the equations are not shown symbolically, which often makes the equations difficult to read, especially for those not involved in the development. However, symbolic equation editors are less often used than spreadsheet software by pharmaceutical researchers. Therefore, although the equations might be more difficult to read, it will be easier to apply and adapt the model, since the user will be more familiar with the software. In addition, during the development of the model, equations can be easily clarified by adding comments, descriptions, and pictures, as we did with the presented model.

Compared to commercial software packages, open source software allows those with less extensive budgets to be able to use the model as well. The difference between the open source software, such as Libreoffice or OpenOffice, and commercial software, such as Microsoft Office, is rather small. Anyone that has experience with any of these packages will be able to find their way in the other packages as well. However, during the development of the model a difference was found in the way iterative calculations are handled that does make the modeling in the open source packages slightly more challenging. First, the number of iteration steps in Excel is limited to 10000, whereas the limit in OpenOffice and Libreoffice is only 1000. Therefore, it can be more challenging to let the iterative calculations converge in the open source software. Secondly, there is a difference in the handling of iteration convergence. All software options allows one to choose a maximum change value, which is the maximum amount a value is allowed to change between iterations before it is considered converged. However, in Excel this value will only determine whether or not the iterative calculation will stop before the iteration step limit is reached. If convergence is not reached, the last calculated values are shown. In the open source alternatives the value is only shown when convergence is reached. When it is not reached, the cell will return an error value. Although this does make it clear that the iterative calculation did not finish, it severely hinders the debugging of such convergence issues, since the source of the problem cannot be identified. Although most convergence issues have been solved during the testing and use of the model, sometimes the model will not converge when the input is changed radically or when the input would result in outlet conditions with a relative humidity close to 100%. In most situations where the model has a problem to converge, simply forcing a recalculation will suffice. However, in case this is not sufficient, additional tips on how to solve or prevent convergence issues are discussed in the short guide accompanying the model (File S3).

When comparing the modeled outlet temperature to the experimental results, it was shown that the model was able to predict the outlet temperature of the spray dryer quite well (Figures 3–4). In addition to the results with the B-290 spray dryer, a good prediction of the outlet temperature of a B-90 spray dryer was found, showing that the model can be used for other types of spray dryers as well. The adaptation of the model was found to be rather straightforward, mainly due to the aspirator flow that was reported in the proper units by the B-90 spray dryer (L/min instead of percentage). Although some knowledge of spreadsheet software is required to be able to change the calculation of the aspirator flow when the spray dryer does not report the proper units, this will generally not be a major problem for most pharmaceutical scientists.

Although the modeled outlet temperature shows only a minor underestimation (<1°C), and a good precision to around 1°C, some of the experimental values that were used to validate the model were found outside the 95% confidence interval (Figure 4). Therefore, it could be concluded that the 95% confidence interval shown here is simply too small, which is most likely true. However, the main reason for the underestimation of the confidence interval is most likely the small dataset that was used to fit the model and calculate the confidence interval. This was deliberately done to show the flexibility of the model and how quick the model can be fitted to a particular spray dryer, while still obtaining a reasonable accuracy. When we consider the small mean (absolute) differences that were found, it can only be concluded that the model is able to predict the outlet temperature quite accurately even when only a small dataset of 8 measurements is used to fit the model to the spray dryer.

To enable the application of the model to the spray drying of amorphous materials, as is often used for stabilizing biopharmaceuticals, the model was extended to calculate the relative humidity. The relative humidity calculation in the model was shown to give a good estimate of the experimentally determined outlet condition (Figure 5). Especially the mean (absolute) difference showed that the model has no bias to under- or overestimate the relative humidity, and that the modeled values are fairly precise to around 1.2% RH. However, the 95% confidence interval that was determined from the outlet temperature dataset used for fitting the model, was clearly too small (data not shown). In addition, even when the confidence interval of the fitting parameter was calculated from the relative humidity instead of the outlet temperature, the resulting confidence interval of the modeled relative humidity would be too small (data not shown). This is most likely due to the physical relation between the fitting parameter, d_air_, the outlet temperature, and the relative humidity. Whereas the outlet temperature is directly influenced by the thickness of the boundary layer for heat conduction (d_air_), the relative humidity is only influenced indirectly. Therefore, d_air_ might not be a suitable fitting parameter for the relative humidity and therefore also does not give a suitable confidence interval based on the method described by Kemmer et al. [16]. However, using a more general method that directly calculates the 95% confidence interval of the modeled relative humidity based on the experimental values was shown to include all experimental values. Although the interval of 3.1% RH may seem rather large, the difference in moisture content of trehalose or inulin at the outlet is only about 0.6%. This translates into a difference of the glass transition temperature of 4–8°C. Although this could be the difference between a rubbery or a glassy sugar, the difference between the outlet temperature and the glass transition temperature of the sugar should be much larger to minimize the molecular mobility and therefore maximize protein stability [21]. Therefore, the 95% confidence interval of 3.1% RH should be sufficient for relative humidity sensitive operations, such as protein stabilization.

Although not clearly pronounced in Figure 5, many of the higher deviations from the modeled relative humidity were found in cases where the outlet temperature is low. At these low temperatures, the relative humidity is much more sensitive to small deviations in moisture content of the humid air. Especially the assumptions made regarding the inlet conditions of the air coming from the dehumidifier (0°C and 100% relative humidity) are of great influence. When the inlet condition was changed by only 1 or 2°C, the relative humidity could change up to 2% RH. The same could also be the said for the temperature difference between that reported by the relative humidity sensor and the B-290 spray dryer. Although the difference was around 2°C, the influence on the relative humidity could be significant. However, this was tested by fitting the model to the outlet temperature measured with the relative humidity probe and the difference was found to be minor (up to 3% RH, with an average difference of 1% RH). In addition, since the relative humidity describes the moisture content in the air, the liquid feed flow and aspirator flow are of importance. Although the liquid feed was facilitated by a roller pump, the pulsation in the liquid feed flow is not expected to have influenced the relative humidity measurement, since the measurement was done over a period of 30 seconds; much longer than the pulsation period, which was around 2 seconds. However, the aspirator flow had to be determined with the flow rate – pressure drop relationship, due to the aspirator flow being expressed in percentage instead of units for flow rate. The equipment that was used, only allowed the pressure drop to be measured at a flow up to 150 L_n_/min, whereas the aspirator flow that was estimated using this method varied between 190 and 370 L_n_/min, which is outside the reference measurement range. However, the error (if any) must be quite small, since a wrong estimation would result in a deviation of the relative humidity for all experiments, which was not apparent in the results, as shown by the small mean difference (0% RH).

Although the relative humidity was found particularly useful for production of amorphous materials by spray drying, the relative humidity was also used as a variable to improve the output of the model. Without information on relative humidity, it remains unknown how much liquid can actually be evaporated. Therefore, the assumption was made that all the liquid fed into the spray dryer was evaporated. This also meant that when the liquid feed flow in the model was set higher than what could in practice be evaporated due to relative humidity limitations at the outlet, the model would simply use this information and return an outlet temperature based on unrealistic circumstances. In other words, the model would predict a combination of liquid feed flow and outlet temperature that would in practice result in a wet product. However, since the relative humidity calculation was added to the model, the assumption that all liquid is evaporated was no longer necessary. Instead, the relative humidity could be coupled to the amount of liquid evaporated to cap the relative humidity at 100%. If the calculated relative humidity would be higher than 100%, the model would simply reduce the amount of evaporated liquid until a relative humidity of 100% is reached. Therefore, the model will no longer predict a relative humidity above 100%, and does not overestimate the amount of liquid evaporated in case too much liquid is sprayed into the modeled spray dryer. Although such conditions are very unlikely to be sought after in a spray drying process, the addition of such calculations does help in reducing the amount of misinformation that could otherwise be obtained by using the model. In addition to the liquid feed flow, knowing the relative humidity also allows one to calculate the adiabatic saturation temperature, which is close to the wet bulb temperature and could be used as an indication of the product temperature during evaporation of the liquid prior to crust formation. However, no experiments have been performed to validate these additions. Besides the relative humidity, extending the model was also found to be quite useful for less common spray dryer configurations. For example, spray drying is usually performed on a single liquid solution. However, there are many interesting applications in which two separate solutions are introduced into the spray dryer to form a mixture with the use of a 3 or 4-fluid nozzle [31], [32]. Therefore, a second liquid stream was added to the model, which enabled the prediction of the outlet conditions depending on the ratio of the two liquid feed flows.

Finally, the good estimate of the relative humidity at the outlet of the spray dryer enabled the prediction of dried product conditions. By combining the modeled outlet conditions with DVS and DSC measurements, the influence on the yield of the dried product could be predicted. However, one could argue that the outlet temperature of the spray dryer did not correspond to the temperature at which the DVS measurements were performed. Therefore, the moisture content of trehalose and inulin that was calculated could deviate significantly from the actual value. However, DVS isotherms of trehalose measured at 45 and 65°C indicated that the moisture content did not change with temperature (data not shown). Therefore, DVS measurements at 25°C could be used to calculate the glass transition temperature of trehalose and inulin at the spray dryer outlet temperature between 32 and 39°C. When the glass transition temperature of trehalose or inulin at the modeled outlet conditions was predicted to be lower than the outlet temperature, the yield was lower than when the glass transition temperature was predicted to be higher than the outlet temperature. This shows that the model allows a wide variety of assessments to be made before spray drying experiments are performed, when the model is combined with other analytical techniques.

## Conclusion

A spray dryer model is presented that is both clear to understand for experienced and novice users, and also readily available online for everyone. Due to the use of open source software for the development, free use of the model is ensured. It was shown that the model can predict the outlet conditions very well for a wide range of spray dryer settings, which enables the user to move from a trial-and-error approach to a quality-by-design approach. In addition, the model can easily be adapted for other types of spray dryers and combined with other analytical techniques such as DSC and DVS to get a better indication of the product properties prior to spray drying.

## Supporting Information

File S1 **Spray dryer model for use with open source software.**
(ODS)

File S2 **Spray dryer model for use with Excel version 2003 and higher.**
(XLS)

File S3 **Short guide for the developed spray dryer model.**
(DOC)
