# Supplementary material for: A User-Friendly Model for Spray Drying to Aid Pharmaceutical Product Development
Source: PLoS One. 2013 Sep 9;8(9):e74403. doi: 10.1371/journal.pone.0074403 (PMC3767666; doi:10.1371/journal.pone.0074403)
Supplement: File S3 — Short guide for the developed spray dryer model. (DOC) [file pone.0074403.s003.doc]

**Spray dryer model – a short guide**

Two versions of the spray dryer model are available for download: one suitable for Excel 2003 and higher (xls file, Supporting Information 2) and one for open source software such as Libreoffice and OpenOffice.org (ods file, Supporting Information 1). The excel file might not be compatible with versions of excel prior to 2003, while the open source file was tested in Libreoffice 3.4 and OpenOffice.org 3.3.

Before working on the model, make sure the following settings are enabled and set in the software package of choice. The iteration option has to be switched on for the model to work, the number of iterations should be set at 1000, and the convergence requirements should be set at 10^-15^. For handling multiple experiments it can be advantageous to also set the calculation to manual.

The model is supplied with three separate sheets. The first two sheets are the models for the B-290 and B-90 spray dryer, as used for the aticle that accompanied this model. When a model needs to be adapted for other spray dryer configurations, a sheet can be copied and renamed for the new model version. The third sheet contains a collection of parameters and options for dropdown menu’s, which is used by the model sheets. Although any changes made to the existing content of the third sheet will reflect back to the model sheets, there is room for additional information, for example regarding the properties of solvents.

By default, the sheets are locked to prevent any accidental changes that could render the model useless. To be able to adapt the model, the respective sheet should be unlocked and preferable afterwards locked again. This can be done by selecting “sheet” in the protection menu and entering the password “EditModel” (without quotation marks). This will unlock or lock all the fields in a sheet that contain an equation or fixed value for editing.

When a model is chosen and/or adapted to the needs of the spray dryer in use, the process parameters and settings can be entered into the model. The input fields for the required information is highlighted in yellow, while any other information should not be edited. The process settings can be found below the process properties section. Although a given spray drying experiment can be run one at a time, it is also possible to calculate multiple experiments at once. To achieve this, the entire row containing the process settings, results, and calculations can be copied to subsequent rows. Each row will then present a separate experiment. This will make it easier to visualize the output, e.g. using graphs.

Although during the development most convergence issues were solved, they may still occur. Depending on whether the Excel or open source file is chosen, the handling of convergence issues will be handled differently. In Excel a result will be shown, whether it is converged or not, while in the open source software and error (error 523) will be shown instead of the results. To tell whether a result is converged in Excel, the calculation can be started again by pressing *F9*. If the values are still changing significantly during the iterative calculation, the result is not converged. Issues with convergence can have several causes and while some can be prevented or fixed, others simply cannot. Unresolvable convergence issues were only encountered during development of the model when the input parameters were outside of the practical range. When the input parameters are within reasonable bounds, a convergence issue can occur due to strongly changed input parameters compared to the previously calculated setting. Most of the times it suffices to simply recalculate the result by forcing the recalculation. In Excel this can be done by pressing *F9*, while in the open source software *CTRL+Shift+F9* is used. In case a forced recalculation is not sufficient, it might help to change the input parameters in increments that can be calculated, until the desired setting is reached.
